# Crystallography Across Temperature Extremes: Insights into the Conformational Landscapes of Human Kinases

**DOI:** 10.1063/4.0001107

**Published:** 2025-10-27

**Authors:** Michael C Thompson

**Affiliations:** University of California, Merced, Merced, CA, USA

## Abstract

Protein kinases are a large family of enzymes that regulate diverse cellular processes by transferring phosphate groups from ATP to their protein substrates. The enzymatic kinase domain (KD) represents a conserved fold, and KD phosphorylation, as well as interactions with cis-regulatory elements and other allosteric effectors, modulate the populations of catalytically active and inactive states present in the KD conformational ensemble. In this presentation, I will briefly describe two studies where performing X-ray crystallography across "extreme" temperature ranges has provided new insight into the conformational landscapes of two human kinases. In one study, we used determined crystal structures of CDK2, a kinase that regulates the cell cycle and is considered a "white whale" of drug discovery, across a broad range of temperatures from cryogenic to physiological, to identify inactive conformations that could be targeted to create more selective inhibitors that avoid off-target effects. In the other study, we determined crystal structures of CLK1 at physiological extremes to understand how this kinase acts as an acute body temperature sensor. Collectively, these studies demonstrate how both physiological and non-physiological temperature perturbations can be used to explore the complex behavior of dynamic proteins.

